# Crossroads of well-being: oral health, neurology, neuronutrition and healthy aging

**DOI:** 10.1515/med-2026-1515

**Published:** 2026-09-28

**Authors:** Ksenia Popovskaya, Anastasiia Badaeva, Anastasiia Kosareva, Viacheslav Novikov, Andrey Danilov, Alexey Danilov, Vittorio Calabrese, Luay Rashan, Gaetano Isola, Alexander Sobin, Bekhruz Rasamatov, Vladimir Kotenev, Eva Gosteeva, Daria Archakova, Ekatherina Vitish, Artem Zacharyan, Margarita Sachkova, Alena Popova, Vlada Korlykhanova, Vitalina Rudenok

**Affiliations:** Department for Nervous Diseases, I.M. Sechenov First Moscow State Medical University of the Ministry of Health of the Russian Federation (Sechenov University), Moscow, Russia; Department for Pathological Physiology, I.M. Sechenov First Moscow State Medical University of the Ministry of Health of the Russian Federation (Sechenov University), Moscow, Russia; Department of Biomedical and Biotechnological Sciences, University of Catania, Catania, Italy; Biodiversity Unit, Dhofar University, Salalah, Oman; Department of General Surgery and Surgical Medical Specialties, University of Catania, Catania, Italy; National Medical Research Center for Hematology, Moscow, Russia; Department for Dentistry, I.M. Sechenov First Moscow State Medical University of the Ministry of Health of the Russian Federation (Sechenov University), Moscow, Russia; Department for Gastroenterology and Hepatology, I.M. Sechenov First Moscow State Medical University of the Ministry of Health of the Russian Federation (Sechenov University), Moscow, Russia; Institute of Leadership and Healthcare Management, I.M. Sechenov First Moscow State Medical University of the Ministry of Health of the Russian Federation (Sechenov University), Moscow, Russia

**Keywords:** oral microbiome, neurodegeneration, systemic inflammation, neuronutrition, periodontitis, oral–gut–brain axis

## Abstract

Oral health is increasingly recognized as a critical component of systemic and neurological resilience during aging. In particular, the oral microbiome may represent a modifiable interface linking periodontal inflammation, neuroimmune activation, and the risk of neurodegenerative disorders.Emerging evidence increasingly supports a link between oral microbiome dysbiosis and the pathogenesis of neurodegenerative disorders. Chronic inflammatory conditions, such as periodontitis, are associated with systemic inflammation, which may contribute to neuroinflammation. Key oral pathogens can translocate into the systemic circulation, compromise the integrity of the blood–brain barrier, and activate microglia. Mechanisms linking oral microbiome dysbiosis to neurodegeneration include systemic inflammation mediated by pro-inflammatory cytokines, direct bacterial invasion of the central nervous system, and modulation of the oral–gut–brain axis through alterations in the gut microbiota and neuroimmune interactions. Personalized neuronutritional strategies, including dietary intake and supplementation with polyphenols, may improve oral health and reduce systemic inflammation. The interdisciplinary integration of neurology, dentistry, and neuronutrition offers new opportunities for the prevention and management of neurodegenerative disorders. Promising approaches include the development of early diagnostic biomarkers of oral dysbiosis and targeted interventions aimed at restoring microbial homeostasis. Further research is needed to clarify causal relationships and optimize strategies for modulating the oral microbiome to preserve cognitive function.

## Introduction

The oral microbiome is a highly diverse microbial ecosystem, comprising bacteria, fungi, archaea, and viruses, that is crucial for maintaining oral health and influencing systemic physiology. Distinct oral niches (tooth surfaces, gingival crevices, the tongue, and other oral habitats) harbor unique microbial communities that function in symbiosis with the host to prevent pathogen colonization and modulate immune responses. Factors such as diet, oral hygiene, smoking, and systemic health can disrupt this balance, leading to dysbiosis, which not only causes oral diseases such as dental caries and periodontitis but may also have far-reaching effects on systemic and neurological health [1].

In recent years, the oral microbiome has emerged as a relevant biological interface between environmental exposures, immune regulation, metabolic signaling, and brain health [2]. The oral cavity is continuously exposed to dietary components, pathogens, hygiene-related factors, and systemic inflammatory conditions, making it a dynamic site where local microbial imbalance may translate into broader biological consequences. In this context, oral dysbiosis should not be considered only a determinant of dental caries or periodontal disease, but also a potential contributor to systemic inflammatory tone, endothelial dysfunction, and altered immune–brain communication [3]. This concept is particularly important in aging, when immunosenescence, reduced epithelial and vascular barrier integrity, and cumulative inflammatory burden may increase susceptibility to neurodegenerative processes. Therefore, the maintenance of oral microbial homeostasis may represent a clinically accessible and potentially modifiable strategy to support healthy aging and reduce neuroinflammatory vulnerability.

**Figure 1:**
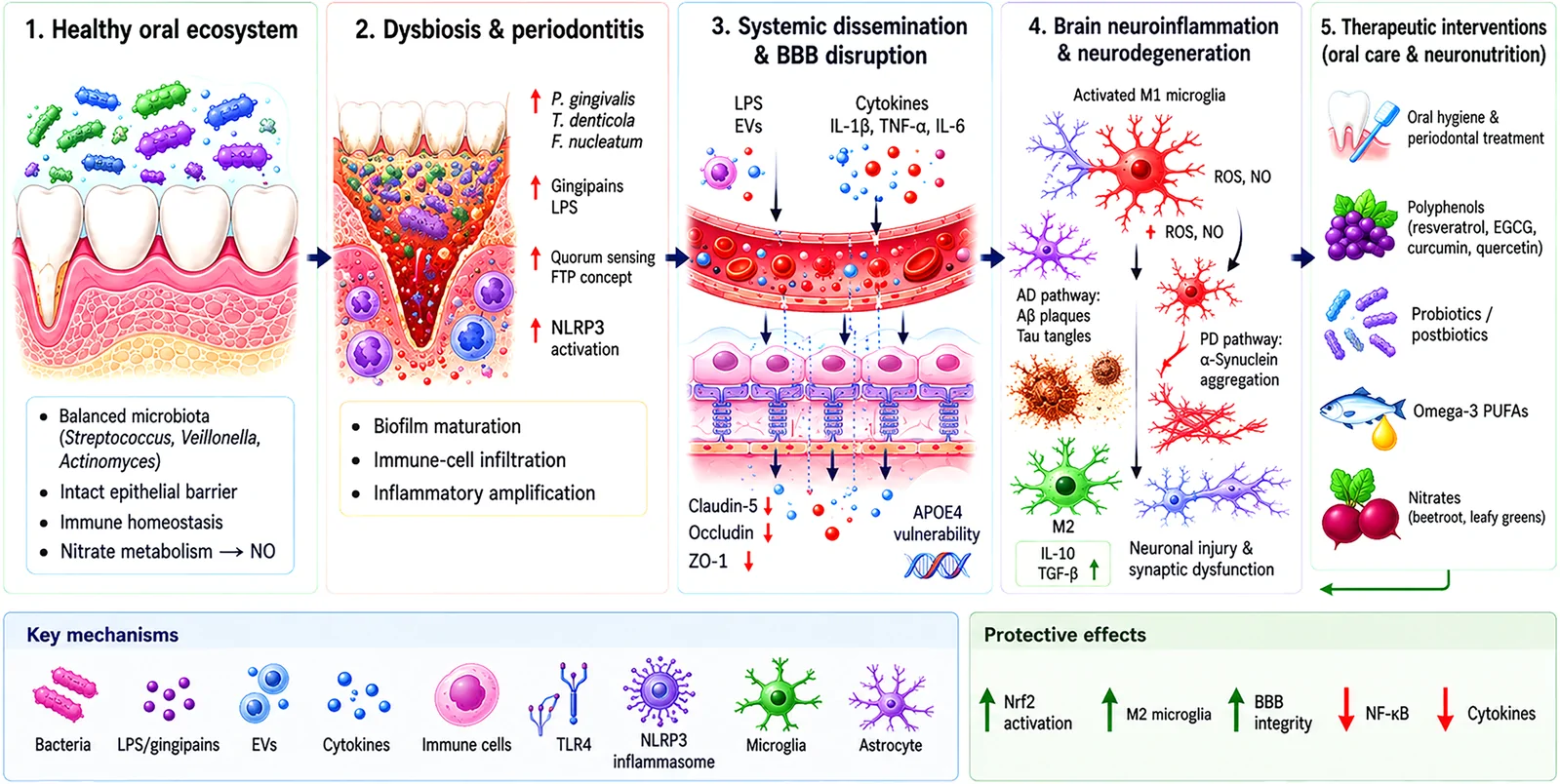
From oral eubiosis to neurodegeneration: mechanistic integration of the oral–brain axis.

Chronic periodontal infection and the resulting inflammatory response have been linked to neurodegenerative processes (Figure 1). Proposed mechanisms include direct bacterial invasion of the brain, systemic dissemination of pro-inflammatory cytokines and endotoxins that trigger neuroinflammation, and molecular mimicry that contributes to neuropathology [3]. Epidemiological studies have demonstrated associations between long-standing periodontitis and an increased risk of cognitive decline. A common denominator underlying these mechanisms is chronic inflammation: oral dysbiosis creates a state of sustained peripheral inflammation that may compromise the blood–brain barrier, activate microglia, and potentiate neurodegeneration [3]. These findings suggest that preserving oral microbial homeostasis through good oral hygiene and periodontal care is not only essential for oral health but may also represent a modifiable factor in reducing the burden and progression of neurodegenerative diseases.

The schematic illustrates the proposed mechanistic continuum linking oral microbial homeostasis to neuroinflammation and neurodegeneration. (1) Under physiological conditions, the healthy oral ecosystem is characterized by a balanced microbial community dominated by commensal genera (e.g., Streptococcus, Veillonella, and Actinomyces), intact epithelial barrier function, immune homeostasis, and physiological nitrate metabolism contributing to nitric oxide (NO) production. (2) Ecological disruption promotes periodontal dysbiosis, characterized by the expansion of keystone pathogens, including *Porphyromonas gingivalis, Treponema denticola, and Fusobacterium nucleatum*. Their virulence factors, including lipopolysaccharide (LPS) and gingipains, together with quorum sensing, polymicrobial interactions, and NLRP3 inflammasome activation, amplify local inflammation. (3) Bacterial products, extracellular vesicles (EVs), and pro-inflammatory cytokines (IL-1β, IL-6, and TNF-α) disseminate systemically, contributing to blood–brain barrier (BBB) dysfunction through disruption of the tight junction proteins claudin-5, occludin, and ZO-1; this process may be further enhanced by APOE4-associated barrier vulnerability. (4) Within the central nervous system, persistent peripheral inflammatory signals promote microglial activation and oxidative stress, leading to sustained neuroinflammation, synaptic dysfunction, and pathological protein aggregation, including amyloid-β deposition, tau pathology, and α-synuclein aggregation. (5) Potential intervention strategies include periodontal therapy, optimization of oral hygiene, neuronutritional approaches based on polyphenols, probiotics/postbiotics, omega-3 polyunsaturated fatty acids, and dietary nitrate sources, which may attenuate neuroinflammation by preserving BBB integrity, modulating microglial activation, suppressing NF-κB signaling, reducing cytokine production, and activating cytoprotective pathways such as Nrf2.

Abbreviations: BBB, blood–brain barrier; EVs, extracellular vesicles; IL, interleukin; LPS, lipopolysaccharide; NF-κB, nuclear factor kappa B; NLRP3, NOD-like receptor family pyrin domain-containing 3; NO, nitric oxide; ROS, reactive oxygen species.

This review was conducted according to the principles of a structured narrative review. The literature was identified through searches of the PubMed, Scopus, and Web of Science databases using the following search terms and their combinations: “oral microbiome,” “oral dysbiosis,” “periodontitis,” “neurodegeneration,” “Alzheimer’s disease,” “Parkinson’s disease,” “blood–brain barrier,” “neuroinflammation,” “polyphenols,” neuronutrition,” and “oral–gut–brain axis.” Eligible studies included peer-reviewed original research articles, systematic reviews, meta-analyses, and clinical trials published primarily between 2015 and 2025, while seminal earlier studies were included where appropriate. Studies were excluded if they lacked sufficient methodological detail, were not published in English, or did not address the relationship between oral health and neurological outcomes. This review synthesizes epidemiological evidence, mechanistic studies of bacterial translocation and neuroimmune signaling, and interventional research to provide a structured overview of the current evidence linking oral dysbiosis with central nervous system (CNS) inflammation and neurodegeneration.

## The burden of neurodegenerative disorders

Neurodegenerative diseases (NDs) are a heterogeneous group of complex disorders characterized by progressive degeneration of the nervous system, particularly highly differentiated structures such as neurons, and represent a major public health challenge worldwide [4]. Epidemiological studies assessing mortality, prevalence, and years lived with disability attributable to NDs between 1990 and 2021 included approximately 3.4 billion individuals. According to these analyses, the global incidence of NDs has increased by 18.2 %, largely owing to increased life expectancy, whereas mortality per 100,000 population has decreased by 33.6 %. Nevertheless, the overall burden of neurodegenerative diseases continues to rise each year [5]. Among these disorders, Alzheimer’s disease (AD), Parkinson’s disease (PD), amyotrophic lateral sclerosis (ALS), and Huntington’s disease (HD) deserve particular attention.

### Alzheimer’s disease (AD)

The accumulation of amyloid-beta (Aβ) and hyperphosphorylated tau protein is considered a central pathological hallmark of AD. Amyloid-beta plaques and neurofibrillary tangles composed of hyperphosphorylated tau accumulate in the brain, particularly in the hippocampus, impairing short-term memory during the early stages of the disease. As AD progresses, long-term memory also becomes affected, ultimately leading to profound cognitive decline [6].

Emerging evidence directly links these classical pathological hallmarks to oral dysbiosis. *P. gingivalis* and its cysteine protease virulence factors, gingipains, have been detected in post-mortem AD brain tissue, while experimental inhibition of gingipains significantly reduced amyloid-beta burden and tau hyperphosphorylation in murine models. In addition, *P. gingivalis*-derived lipopolysaccharide (LPS) activates TLR4/NF-κB signaling in microglia, triggering pro-inflammatory cascades that promote both amyloid precursor protein cleavage and tau kinase activation. *T. denticola* has likewise been proposed to reach the central nervous system (CNS) via retrograde transport along cranial nerve pathways, and its protease complex, dentilisin, may directly impair neuronal integrity [6]. Collectively, these findings suggest that the amyloid and tau pathology central to AD may be amplified by chronic oral infection, supporting periodontal disease as a potentially modifiable risk factor in AD prevention.

### Parkinson’s disease (PD)

PD is the fastest-growing neurodegenerative disorder worldwide, and its prevalence is expected to continue increasing through 2030. The cardinal motor manifestations include bradykinesia, resting tremor, rigidity, and postural instability [7]. Non-motor manifestations are equally important and include sensory disturbances (pain and hyposmia), cognitive and psychiatric symptoms (depression, anhedonia, and behavioral disorders), and autonomic dysfunction (orthostatic hypotension, constipation, and urogenital dysfunction). Many of these non-motor symptoms emerge during the prodromal phase and are associated with α-synuclein aggregation [8].

Importantly, the prodromal non-motor features of PD – including hyposmia, constipation, and autonomic dysfunction – parallel established routes of oral–CNS communication. α-Synuclein aggregation, the neuropathological hallmark of PD, has been identified in the enteric nervous system years before the onset of motor symptoms, supporting a gut-to-brain prion-like propagation model in which oral dysbiosis may represent an upstream trigger. LPS and short-chain fatty acids associated with oral pathogens such as *P. gingivalis* and *F. nucleatum* may promote systemic and neuroinflammation, thereby accelerating α-synuclein misfolding through activation of the microglial NLRP3 inflammasome [2], 8]. Moreover, periodontitis-associated hyposalivation, frequently observed in patients with PD due to autonomic dysfunction, may further destabilize the oral microbiome, creating a self-reinforcing cycle of oral dysbiosis, systemic inflammation, and progressive neuroinflammation.

### Amyotrophic lateral sclerosis (ALS)

ALS is a progressive neurodegenerative disease affecting both upper and lower motor neurons, with peak incidence occurring between 60 and 79 years of age. Clinically, ALS is characterized by progressive skeletal muscle weakness, eventually leading to respiratory failure due to diaphragmatic paralysis. The disease exhibits considerable phenotypic heterogeneity, including bulbar-onset, cervical-onset, lumbar-onset, respiratory-onset, flail arm, flail leg, primary lateral sclerosis, and progressive muscular atrophy phenotypes. Pathogenic variants in C9orf72, TARDBP, SOD1, and FUS are among the most frequently identified genetic causes. Median survival following diagnosis is generally 2–4 years [9].

Although the association between the oral microbiome and ALS remains less well characterized than in AD or PD, shared inflammatory mechanisms suggest a plausible contributory role. ALS is characterized by pronounced activation of microglia and astrocytes together with elevated systemic concentrations of pro-inflammatory cytokines, including IL-6 and TNF-α – the same mediators induced by periodontal pathogens through activation of NF-κB and the NLRP3 inflammasome. Furthermore, gut dysbiosis, which may be partially influenced by oral bacterial translocation through the oral–gut axis, has been reported in ALS cohorts and has been associated with disease progression. Future studies investigating the contribution of *P. gingivalis* virulence factors to TDP-43 aggregation and SOD1-mediated oxidative stress may identify novel preventive targets at the oral–neurological interface [2], 9].

### Huntington’s disease (HD)

HD is an autosomal dominant neurodegenerative disorder characterized by progressive motor dysfunction, cognitive decline, and psychiatric manifestations. The disease results from expansion of the CAG trinucleotide repeat within the HTT gene, leading to the production of mutant huntingtin protein. Neuropathological changes primarily affect the striatum and cerebral cortex. Early manifestations include subtle involuntary movements and cognitive impairment, whereas advanced disease is characterized by severe motor disability, dysphagia, and dementia. At present, treatment remains symptomatic, and no disease-modifying therapy has been established [10].

Despite substantial advances in understanding the genetic basis of neurodegenerative diseases, their pathogenesis remains incompletely understood and is believed to involve complex interactions among genetic, epigenetic, environmental, and inflammatory factors. Increasing evidence suggests that the oral microbiome may represent one such environmental contributor.

The predominant healthy oral microbiota include members of the genera Gemella, Granulicatella, Streptococcus, and Veillonella, which contribute to the maintenance of oral homeostasis. In patients with AD, cognitive impairment often compromises oral hygiene, increasing the prevalence of dental caries and periodontitis. Periodontal disease promotes the production of pro-inflammatory cytokines, including IL-1β, IL-6, and TNF-α [11]. These cytokines, particularly IL-1β and IL-6, have been proposed to contribute to amyloid plaque formation and disease progression [12]. Similarly, impaired motor function in PD frequently compromises oral hygiene, predisposing patients to dental caries and periodontitis [11]. Neuroinflammation is considered a central mechanism in PD pathogenesis, with activated microglia producing IL-6, IL-1, TNF-α, IL-10, IL-12, IL-8, IL-17A, and interferon-γ. These cytokines also regulate chemokine production, facilitating monocyte infiltration into the brain in response to α-synuclein aggregation [13].

Collectively, these observations highlight the potential contribution of oral dysbiosis to neurodegenerative diseases and emphasize the importance of elucidating the molecular mechanisms linking the oral microbiome with neuroinflammation and neurodegeneration [4].

## The oral microbiome and neurodegeneration

### Composition and diversity of the oral microbiome

The human oral cavity harbors one of the most diverse microbiomes in the body, consisting of more than 700 bacterial species across approximately seven phyla, together with fungi, archaea, and viruses [14], [15], [16]. This complex microbial community typically exists in a balanced state (eubiosis) that contributes to oral homeostasis. The core microbiota (e.g., *Streptococcus*, *Veillonella*, and *Actinomyces* spp.) is relatively conserved among individuals; however, substantial interindividual variability exists among less abundant taxa and at the strain level [14], [15], [16]. Advances in 16S rRNA gene sequencing and metagenomics have expanded our understanding beyond bacteria, revealing a resident oral virome and mycobiome [15], 16].

Microbial diversity is influenced by distinct habitats within the oral cavity. The tongue, teeth, gingival crevice, cheeks, palate, and saliva each support unique microbial communities [14]. Healthy oral biofilms generally maintain a dynamic equilibrium in which commensal microorganisms predominate and suppress the overgrowth of potential pathogens. High microbial diversity is commonly associated with oral health, whereas alterations in community composition (dysbiosis) predispose to disease [14], 15]. Importantly, the oral microbiome is dynamic rather than static. It undergoes succession from infancy, when initial colonizers originate from maternal and environmental sources, through adulthood and continues to change in response to diet, oral hygiene, and other environmental factors [16]. Overall, the remarkable diversity and resilience of the oral microbiome underpin its essential roles in maintaining oral ecosystem stability and supporting host physiological functions.

### Key niches in the oral cavity

Microorganisms within the oral cavity are not uniformly distributed; instead, they colonize distinct ecological niches and form biofilms on specific oral surfaces.

Dental plaque (on tooth surfaces) and the tongue dorsum are among the most densely colonized sites, each harboring characteristic microbial communities. For example, dental plaque biofilms, particularly within the subgingival crevice, favor anaerobic genera such as *Porphyromonas*, *Tannerella*, and *Fusobacterium*, whereas the oxygen-rich tongue surface supports a greater abundance of aerobic microorganisms and acidogenic streptococci [14], 17].

The keratinized gingiva and buccal mucosa harbor distinct microbial communities, and the microbial profiles of dental plaque, the tongue, and the gingiva differ considerably from one another [14]. Saliva, although not a permanent surface itself, serves as a vehicle for microbial dissemination between oral niches and reflects the overall composition of the oral microbiome.

Environmental factors within each niche – including oxygen availability, salivary flow, pH, and host receptor expression – determine which microorganisms can successfully colonize these habitats [14]. For example, supragingival plaque is exposed to saliva and moderate oxygen concentrations, favoring facultative bacteria such as *Streptococcus* and *Actinomyces*, whereas the subgingival pocket provides an anaerobic, protein-rich environment that supports proteolytic Gram-negative bacteria [16]. The papillary architecture of the tongue also provides protected microenvironments for anaerobic microorganisms despite the generally aerobic conditions, explaining the frequent detection of periodontal anaerobes on the tongue surface [17].

Biofilm architecture further shapes these ecological niches. Microorganisms coaggregate into highly organized communities, exemplified by the characteristic “corncob” structures consisting of central *Corynebacterium* species surrounded by peripheral streptococci, thereby optimizing nutrient utilization and oxygen gradients. Collectively, the oral cavity comprises heterogeneous habitats – including the teeth, gingiva, tongue, saliva, and other mucosal surfaces – each supporting distinct microbial consortia adapted to their local microenvironment [14], 17]. This spatial organization is clinically relevant because dysbiosis within a specific niche, such as the subgingival biofilm during periodontitis, often remains localized initially but may subsequently influence systemic health through microbial dissemination or inflammatory mediators. Understanding these ecological niches is essential for developing targeted preventive and therapeutic strategies aimed at maintaining or restoring a healthy oral microbiome.

### Functions of the oral microbiome in oral and systemic health

The indigenous oral microbiota performs numerous beneficial functions for the host. Under healthy conditions, the oral microbiome provides colonization resistance, whereby commensal microorganisms occupy ecological niches and compete for nutrients, thereby preventing colonization by exogenous pathogens [15], 17]. For example, commensal streptococci produce bacteriocins and regulate local pH, limiting the overgrowth of cariogenic and periodontopathogenic species.

The oral microbiome also contributes to tissue homeostasis through continuous interactions with the host immune system, promoting balanced immune surveillance. Commensal microorganisms induce low-level immune signaling that conditions mucosal immune responses, thereby promoting tolerance toward harmless antigens while maintaining readiness to respond to pathogenic threats [14], 16].

In addition, oral bacteria participate in metabolic processes. A well-established example involves nitrate-reducing bacteria residing on the tongue, which convert dietary nitrate into nitrite and thereby facilitate systemic nitric oxide production, contributing to cardiovascular homeostasis through blood pressure regulation [15]. Consequently, the oral microbiome functions as an important metabolic partner of the host.

Beyond the oral cavity, the oral microbiome exerts systemic effects. Oral bacteria may transiently enter the bloodstream during routine activities such as chewing or tooth brushing, thereby contributing to immune homeostasis beyond the oral cavity [14]. A balanced oral microbiome is associated not only with oral health – including the prevention of dental caries, gingivitis, and periodontitis – but also with a reduced systemic inflammatory burden [15]. Conversely, dysbiosis has been associated with oral diseases and multiple systemic disorders, including cardiovascular disease and adverse pregnancy outcomes, through inflammatory mechanisms and microbial dissemination.

Overall, the oral microbiome plays an essential role in maintaining both oral and systemic health by providing colonization resistance, supporting host metabolism, and interacting closely with the immune system to preserve physiological homeostasis [15], 16]. Disruption of this equilibrium may contribute to both oral and systemic diseases, underscoring the importance of maintaining a healthy oral microbiota.

### Implications of the oral microbiome in neurodegenerative diseases

Epidemiological and clinical studies increasingly support an association between poor oral health, oral dysbiosis, and neurodegenerative disease outcomes.

 Patients with AD have been found to harbor oral bacterial DNA in brain tissue more frequently than cognitively healthy individuals, suggesting translocation of oral microorganisms [18], 19]. Longitudinal studies indicate that a history of chronic periodontitis or tooth loss is associated with an increased risk of cognitive decline and dementia later in life [20], 21]. For example, a recent systematic review and meta-analysis concluded that poor periodontal health, assessed by tooth loss, periodontal pocket depth, or alveolar bone loss, is associated with an approximately 20–23 % increased risk of cognitive decline or dementia [20]. Moreover, patients with AD exhibit higher antibody levels against *P. gingivalis* and other periodontal pathogens, which correlate with increased cerebral amyloid burden and accelerated cognitive decline, suggesting an active contribution of chronic oral infection to disease progression [19]. Although limited, several interventional studies have also suggested that periodontal treatment may improve cognitive function or slow disease progression in patients with AD [16].

The relationship between oral dysbiosis and PD appears to be more complex. Although many patients with PD exhibit poor oral health owing to impaired motor function and altered salivary physiology, the direction of causality remains uncertain. Some observational studies suggest that a history of periodontitis increases the risk of developing PD, whereas recent large-scale analyses and a 2023 meta-analysis found no significant overall association between periodontitis and PD incidence [21], 22]. Nevertheless, patients with PD consistently demonstrate poorer periodontal status and a higher prevalence of periodontitis than healthy controls, indicating that PD itself contributes to oral health deterioration. The relationship is likely bidirectional: chronic peripheral inflammation associated with periodontitis may exacerbate PD pathology, whereas PD-related alterations in salivary secretion, immune regulation, and oral hygiene may further promote periodontal disease. In addition, oral bacteria producing lipopolysaccharide (LPS) and other pro-inflammatory mediators may contribute to the neuroinflammatory processes characteristic of PD. Ongoing studies continue to investigate disease-specific oral microbial signatures and the potential neurological benefits of improved oral healthcare.

Chronic periodontitis has also been associated with an increased risk of vascular dementia and has been proposed as a contributing factor to multiple sclerosis (MS) progression through systemic inflammatory mechanisms [23]. Patients with MS exhibit increased periodontal inflammation and overlapping inflammatory biomarkers, although a direct causal relationship has not yet been established. Furthermore, chronic periodontal inflammation may contribute to vascular cognitive impairment by increasing systemic inflammatory markers, including C-reactive protein and fibrinogen, which adversely affect cerebrovascular health.

Collectively, these findings highlight oral health as an important component of overall health that may influence neurological well-being. Chronic periodontitis and oral dysbiosis, through persistent systemic inflammation and potential microbial dissemination, represent potentially modifiable risk factors for neurodegenerative diseases. Consequently, increasing attention has focused on whether effective periodontal therapy and preventive oral healthcare can reduce the risk or slow the progression of conditions such as AD. Although causality remains under investigation, maintaining good oral hygiene throughout adulthood and later life may represent an important strategy for promoting healthy brain aging [24]. At a minimum, timely diagnosis and treatment of oral diseases may reduce one source of chronic systemic inflammation that contributes to neurodegenerative pathology.

## Mechanisms linking the oral microbiome to neurodegeneration

Growing evidence indicates that oral microbiome dysbiosis, particularly in the context of periodontal disease, contributes to neurodegenerative processes through several interconnected biological mechanisms. These mechanisms include systemic inflammation, microbial dissemination, blood–brain barrier dysfunction, immune activation, and neuroinflammatory signaling.

### Systemic inflammation and immune responses

Current concepts of systemic inflammation associated with oral diseases are largely based on the biological behavior of the dental biofilm. Dental biofilms exist in two functional states: a symbiotic (eubiotic) state that supports oral homeostasis and a dysbiotic state characterized by the expansion of pathogenic microorganisms and persistent inflammation [25]. The transition from eubiosis to dysbiosis represents a critical event in the pathogenesis of periodontitis and has been implicated in numerous systemic inflammatory disorders.

In the healthy oral cavity, the resident microbiota provides colonization resistance, a protective ecological phenomenon consistent with Gause’s competitive exclusion principle, in which commensal microorganisms occupy ecological niches and prevent colonization by opportunistic pathogens [26]. When this protective microbial community is disrupted, opportunistic microorganisms, including *Candida* spp. and *Staphylococcus aureus*, can rapidly proliferate, illustrating the loss of colonization resistance and the establishment of dysbiosis [27].

Understanding the mechanisms that drive the transition from a symbiotic to a dysbiotic microbial community is therefore fundamental to explaining how oral diseases contribute to systemic inflammation. Alterations in colonization resistance are considered one of the earliest events in this transition, ultimately promoting chronic periodontal inflammation and contributing to the systemic inflammatory burden associated with periodontitis [28].

### Introduction to the concepts of dysbiosis origin

During the development of periodontal disease, inflammophilic bacteria exhibit cross-protection and establish polymicrobial synergy. This synergy manifests through multiple complementary mechanisms. In particular, the cooperative formation of multispecies biofilms creates structural conditions that enhance the survival and persistence of the microbial community. For example, experimental studies involving *Streptococcus gordonii*, *F. nucleatum*, and *P. gingivalis* demonstrated that cooperative biofilm formation provides optimal conditions for the survival of all three species [29].

Another important aspect of polymicrobial interactions is the ability of bacteria to alter gene expression when present within a microbial consortium, a phenomenon referred to as the group effect. These interactions are frequently mediated by metabolic cooperation, whereby one bacterial species provides nutrients or metabolites that support the growth and persistence of another. Such nutritionally mediated interactions have been extensively investigated in the model periodontal pathogens *P. gingivalis* and *T. denticola* [30], 31].

Beyond these interactions, polymicrobial synergy can also promote the transition of commensal microorganisms into a more virulent phenotype. Meta-transcriptomic analyses have demonstrated that a substantial proportion of the microorganisms contributing to virulent biomass are species normally associated with a healthy periodontium [32]. This transition from a health-associated commensal to a disease-associated pathobiont is referred to as the Functional Transformation Point (FTP).

These observations can be interpreted within two complementary conceptual frameworks: the dysbiotic cascade concept [33] and the FTP concept, which further expands our understanding of the functional transformation of the oral microbiome during disease progression.

### Mechanisms driving oral dysbiosis

Multiple mechanisms contribute to the transition from oral eubiosis to dysbiosis and the subsequent development of inflammation. Although alterations in the Community Resilience System (CRS) may contribute to this process, they are not always the initiating event, as colonization of the oral cavity by entirely new bacterial species is relatively uncommon. Instead, microorganisms associated with periodontitis are frequently present at low abundance within the healthy oral microbiome [34]. Nevertheless, disruption of colonization resistance becomes increasingly important once inflammation has been established.

Bacterial interactions within oral biofilms are coordinated through quorum sensing (QS) [35]. Successful co-aggregation of microorganisms, which is essential for polymicrobial synergy, depends on precise intercellular communication mediated by signaling molecules including catecholamines, peptides, and other autoinducers [11], [36], [37], [38]. Among the best-characterized signaling molecules are N-acyl homoserine lactones (AHLs) [39]. Although the role of AHLs continues to be actively investigated, experimental studies have demonstrated that lactonase, an enzyme that degrades AHLs, reduces cariogenic biofilm formation while simultaneously promoting the growth of commensal microorganisms [40], 41]. These findings suggest that quorum sensing plays a pivotal role in the development of oral dysbiosis and may indirectly contribute to systemic inflammation.

Within the oral biofilm, microbial influence is not evenly distributed. Certain keystone pathogens, despite their relatively low abundance, exert a disproportionate effect on both the microbial community and the host immune response [28]. *P. gingivalis* represents a prototypical keystone pathogen capable of initiating multiple downstream inflammatory cascades that affect both neighboring microorganisms and host immune cells [32].

A key mechanism underlying this process involves modulation of innate immune signaling. *P. gingivalis* influences the expression of the pro-inflammatory cytokines IL-1β and IL-18 and activates the NLRP3 inflammasome, thereby promoting inflammatory cell death and amplifying local inflammation [42]. In addition, *P. gingivalis* interacts with C5aR1 and TLR2, selectively inducing inflammatory signaling while simultaneously impairing protective phagocytic responses [43], 44]. This immune-evasion strategy is mediated primarily by the gingipains HRgpA and RgpB, which uncouple the protective TLR2–MyD88 signaling pathway from the PI3K pathway, thereby promoting bacterial persistence [43]. Furthermore, *P. gingivalis* suppresses chemokine production through serine phosphatase B, which inhibits transcription of CXCL8 (IL-8) [45].

Inflammation itself promotes the expansion of pathogenic microorganisms while facilitating the functional transformation of commensal bacteria into pathobionts (FTP). This transition represents a critical stage in dysbiosis progression and may be mediated through bacterial extracellular vesicles (exosomes), which transfer virulence-associated factors to neighboring bacteria and host immune cells [42]. As dysbiosis progresses, inflammation further destabilizes the Community Resilience System in accordance with the Ecological Plaque Hypothesis (EPH), creating a self-perpetuating positive feedback loop that reinforces both microbial dysbiosis and chronic inflammation [46]. The systemic consequences of this process are clinically significant. Chronic oral dysbiosis and periodontitis contribute to persistent low-grade systemic inflammation, which has been implicated in the pathogenesis of hypertension [47], diabetes mellitus [48], atherosclerosis [49], and, increasingly, neurodegenerative disorders including AD [50].

### Mouth–gut–brain axis: bacterial metabolites and systemic communication

Approximately 1–1.5 L of saliva is produced daily by the salivary glands. Because the oral cavity and gastrointestinal tract are anatomically continuous, oral microorganisms and their metabolites are continuously transferred to the digestive tract. Consequently, numerous studies have demonstrated that oral microorganisms can influence the composition of the gut microbiota and that bacterial metabolites may affect the brain and systemic physiology through multiple biological pathways [17], 51].

Within the oral–gut axis, hematogenous dissemination through the bloodstream is considered one of the principal routes by which oral microorganisms interact with the gastrointestinal tract [52]. A second proposed pathway involves ingestion. Under specific conditions, such as proton pump inhibitor therapy or antibiotic treatment, oral bacteria may survive passage through the gastrointestinal tract, colonize the intestine, alter gut microbial composition, and promote the production of metabolic endotoxins, pro-inflammatory cytokines, and chemokines through changes in host gene expression [53]. In healthy individuals, the gut microbiota is dominated by members of the phyla *Firmicutes, Bacteroidetes, Proteobacteria*, and *Actinobacteria*, with smaller populations of *Fusobacteria, Cyanobacteria*, archaea, fungi, and viruses [54].

Disruption of either the oral or intestinal microbial community influences the reciprocal interaction between these two ecosystems and contributes to the development of systemic and neurodegenerative diseases. Experimental evidence supporting this relationship has been obtained from animal models in which transplantation of saliva from patients with severe periodontitis into mice significantly altered gut microbial β-diversity. In these studies, members of the *Porphyromonadaceae* and *Fusobacterium* families became enriched, whereas the abundance of *Akkermansia* was reduced compared with control animals [55].

In contrast, direct transfer of intestinal bacteria to the oral cavity appears to be uncommon, with fecal–oral transmission considered the principal route of microbial exchange in the reverse direction [56], [57], [58]. Evidence supporting a direct oral–brain axis remains comparatively limited. Current data suggest that oral bacteria and their metabolites may influence the central nervous system either directly, through the trigeminal, olfactory, or facial nerves and the systemic circulation, or indirectly through oral-induced gut dysbiosis and systemic inflammation [17], 59]. Alterations in bacterial metabolites and immune signaling have also been implicated in the pathogenesis of inflammatory bowel disease, rheumatoid arthritis, pancreatic cancer, and colorectal cancer [55].

Communication along the gut–brain axis occurs through several interconnected pathways. Direct communication is mediated primarily by the vagus nerve, whereas indirect signaling involves microbial metabolites, immune mediators, and endocrine pathways that collectively regulate bidirectional communication between the gut microbiota and the central nervous system. Disruption of any component of this network may contribute to systemic inflammation and neuroinflammation through activation of the innate immune response of microglia, thereby accelerating neurodegenerative processes, including AD [17], 60], 61]. Conversely, central nervous system disorders may alter the composition of the gut microbiota, leading to impaired intestinal immune homeostasis [62].

Collectively, these findings support the concept that the oral–gut–brain axis represents an important biological pathway linking oral dysbiosis with both systemic and neurodegenerative diseases. Alterations in the oral microbiome may therefore induce secondary changes in gut microbial composition, amplify systemic inflammation, and contribute to neuroinflammatory processes.

### Cytokine-mediated pathways, blood–brain barrier disruption, and microglial polarization

Activation of pro-inflammatory cytokines by oral dysbiosis represents one of the central mechanistic pathways linking periodontal disease with neurodegeneration. During dysbiosis, keystone pathogens such as *P. gingivalis*, *T. denticola*, and *F. nucleatum* release lipopolysaccharide (LPS) and other pathogen-associated molecular patterns (PAMPs) into the periodontal tissues and systemic circulation. LPS activates Toll-like receptor 4 (TLR4) on host immune cells, initiating MyD88-dependent NF-κB signaling and promoting the production of IL-1β, IL-6, and TNF-α [42], 43]. In addition, *P. gingivalis* directly activates the NLRP3 inflammasome, resulting in caspase-1-mediated maturation and release of IL-1β and IL-18. Persistent release of these cytokines establishes chronic low-grade systemic inflammation that extends well beyond the oral cavity.

Peripheral inflammatory signals subsequently induce a phenotypic shift in resident central nervous system microglia toward a pro-inflammatory M1-like phenotype. Activation of TLR2/TLR4-dependent NF-κB and MAPK signaling pathways promotes the expression of inducible nitric oxide synthase (iNOS), IL-1β, IL-6, TNF-α, and reactive oxygen species (ROS)-generating enzymes, including NADPH oxidase. Chronically activated M1 microglia release neurotoxic mediators that impair synaptic function, promote tau hyperphosphorylation, and accelerate amyloid-β aggregation through reduced phagocytic clearance [2]. In contrast, the anti-inflammatory M2 phenotype, characterized by IL-10, TGF-β, and neurotrophic factor production, becomes progressively suppressed under conditions of persistent peripheral inflammation associated with chronic periodontitis. Restoration of the M1/M2 balance through targeted oral and systemic anti-inflammatory interventions therefore represents a promising therapeutic strategy for preventing or slowing neurodegeneration.

### Genetic predisposition, nitric oxide signaling, and additional mechanisms

One proposed mechanism linking oral health with cognitive function involves nitric oxide (NO) production through the nitrate–nitrite pathway. Nitric oxide is an essential signaling molecule involved in vascular regulation, neurotransmission, skeletal muscle function, and host antimicrobial defense [63]. Members of the oral microbiome reduce dietary nitrate to nitrite, which is subsequently converted into NO within the circulation and peripheral tissues. In the brain, NO activates soluble guanylate cyclase and functions as both a pre- and postsynaptic signaling molecule.

During aging, endogenous NO production declines because of reduced nitric oxide synthase (NOS) expression together with increased arginase-mediated arginine degradation. This decline has been associated with hypertension, atherosclerosis, and other cardiovascular disorders [64], [65], [66]. Consistent with these observations, decreased concentrations of nitrate and nitrite, established biomarkers of NO bioavailability, have been reported in both the plasma and brain tissue of patients with atherosclerosis [67].

Recent studies have identified *Prevotella intermedia* as a potential microbial predictor of increased genetic susceptibility to dementia in individuals carrying the APOE4 allele [68]. Based on these findings, it has been hypothesized that the balance between the two major pathways of oral nitrate metabolism – denitrification and dissimilatory nitrate reduction to ammonia (DNRA) – may shift toward DNRA in individuals with mild cognitive impairment (MCI), potentially in an APOE4-dependent manner [68]. The APOE4 allele is a well-established genetic risk factor for AD and has also been associated with impaired blood–brain barrier integrity [69]. Furthermore, APOE4 carriers are at increased risk of hypertension, atherosclerosis, and skeletal muscle weakness, all of which have been linked to reduced nitric oxide bioavailability [70], [71], [72], [73]. Collectively, these findings suggest a potential interaction among APOE4 genotype, nitric oxide metabolism, and the oral microbiome during age-related cognitive decline. However, it remains unclear whether specific oral microbial signatures correlate with cognitive performance or whether alterations in the oral microbiome can be detected in cognitively healthy older adults before the clinical onset of dementia.

### The role of specific microbes

#### P. gingivalis

*Porphyromonas gingivalis* (*P. gingivalis*) is a Gram-negative, obligate anaerobic bacterium that forms part of the normal oral microbiota but acts as an opportunistic pathogen under dysbiotic conditions. It possesses multiple virulence factors that contribute to the development and progression of periodontitis [74]. Poor oral hygiene promotes dental biofilm accumulation, creating favorable conditions for the proliferation of *P. gingivalis*. Within the biofilm, *P. gingivalis* expresses several key virulence factors, including lipopolysaccharide (LPS), fimbriae, and gingipains, which facilitate bacterial adhesion, persistence, and immune evasion [75].

Periodontitis is a chronic infectious disease affecting the supporting tissues of the teeth, leading to gingival inflammation, destruction of the periodontal ligament, and progressive loss of alveolar bone. It is estimated that chronic inflammatory periodontal diseases affect 70–98 % of the global population [76]. *P. gingivalis* plays a central role in periodontitis by disrupting host immune responses through its virulence factors. Furthermore, its biofilm promotes the attachment of additional bacterial species, facilitating the establishment of pathogenic polymicrobial communities [76].

*Porphyromonas gingivalis* forms complexes with glyceraldehyde-3-phosphate dehydrogenase (GAPDH) and other bacterial proteins, promoting biofilm maturation and enabling colonization by additional microorganisms that contribute to the development of a dysbiotic oral microbiome [77]. As discussed previously, periodontitis is one of the most prevalent chronic oral diseases and has been associated with disorders affecting the cardiovascular, reproductive, and nervous systems. The pathogenicity of *P. gingivalis* is largely attributable to its virulence factors, including fimbriae, LPS, and gingipains [78]. Gingipains are cysteine proteases that promote immune dysregulation, chronic inflammation, degradation of host defense proteins, and inactivation of immune cells. Numerous studies have demonstrated that gingipains enhance interactions between *P. gingivalis* and other periodontal pathogens, including *Aggregatibacter actinomycetemcomitans*, thereby facilitating bacterial adhesion and biofilm maturation [79], 80]. In addition, *P. gingivalis* adheres efficiently to gingival epithelial cells and degrades extracellular matrix components, promoting periodontal tissue destruction and gingival bleeding [81].

Experimental studies investigating the relationship between *P. gingivalis* and neurodegenerative diseases have demonstrated the presence of *P. gingivalis*-derived LPS in the brains of infected mice, indicating that bacterial products are capable of reaching the central nervous system [82]. LPS stimulates antigen-presenting cells and promotes the production of pro-inflammatory cytokines. Cytokines such as IL-1α and IL-1β can subsequently reach the brain through the systemic circulation, cerebrospinal fluid, and interstitial fluid, thereby influencing central nervous system function [83], 84].

A growing body of evidence also supports a relationship between *P. gingivalis* infection and AD. Numerous studies have demonstrated that chronic periodontal inflammation contributes to blood–brain barrier dysfunction, a characteristic feature of AD. The disease is characterized by extracellular amyloid-β (Aβ) plaque deposition and intracellular neurofibrillary tangles composed of hyperphosphorylated tau protein, both of which correlate closely with cognitive decline. Chronic colonization by *P. gingivalis* promotes persistent systemic inflammation and facilitates the dissemination of bacterial products into the circulation. Under conditions of blood–brain barrier dysfunction, these inflammatory mediators and bacterial virulence factors may access the central nervous system, activate microglia and astrocytes, and promote neuroinflammation, amyloid-β accumulation, and tau pathology [85], 86].

Collectively, these findings indicate that *P. gingivalis* contributes to neurodegeneration primarily through chronic periodontal inflammation, systemic dissemination of bacterial virulence factors, blood–brain barrier dysfunction, and activation of innate immune signaling pathways.

#### A. actinomycetemcomitans

*Aggregatibacter actinomycetemcomitans* (*A. actinomycetemcomitans*) is a Gram-negative, facultatively anaerobic, non-motile bacterium that forms part of the normal oral microbiota but is strongly associated with aggressive forms of periodontitis owing to its potent immunomodulatory properties. Increasing evidence suggests that periodontal disease associated with *A. actinomycetemcomitans* may contribute to systemic inflammatory disorders, including AD [87].

*Aggregatibacter actinomycetemcomitans* has been implicated in several systemic inflammatory conditions through its ability to modulate innate immune responses. Experimental studies indicate that lipopolysaccharide (LPS) derived from *A. actinomycetemcomitans* activates microglia and promotes the production of pro-inflammatory cytokines. In murine models, hippocampal cells exposed to LPS serotypes A, B, and C demonstrated differential inflammatory responses, with serotype B producing the greatest increase in cytokine expression together with enhanced activation of Toll-like receptors TLR2 and TLR4 [88]. These findings suggest that structural differences among LPS serotypes may influence the magnitude of the neuroinflammatory response.

Whether circulating cytokines directly cross the blood–brain barrier remains a matter of ongoing investigation. However, periodontal LPS has consistently been shown to amplify inflammatory signaling within the central nervous system. Mixed hippocampal cultures stimulated with purified *A. actinomycetemcomitans* LPS exhibited increased cytokine mRNA expression together with elevated production of amyloid-β1–42 (Aβ1–42), supporting a mechanistic link between periodontal inflammation and AD pathology [88].

Collectively, available evidence indicates that periodontal pathogens, particularly *A. actinomycetemcomitans* and *P. gingivalis*, contribute to chronic systemic inflammation and may participate in the pathogenesis of diabetes mellitus, cardiovascular disease, and neurodegenerative disorders through activation of innate immune pathways [89].

#### *Tannerella forsythia, T. denticola*, and *Streptococcus*

*Tannerella forsythia* is a Gram-negative periodontal pathogen characterized by a unique protein O-glycosylation system and multiple virulence factors that contribute to periodontal tissue destruction [90]. One of its most important pathogenic characteristics is its high sialidase activity. *T. forsythia* expresses the sialidase NanH, which cleaves terminal sialic acid residues from host glycoproteins, thereby facilitating bacterial adhesion, invasion of epithelial cells, and persistence within the periodontal environment [91].

*Treponema denticola* is a Gram-negative, obligate anaerobic spirochete and another major periodontal pathogen. Its high motility enables efficient invasion of host tissues, whereas its proteolytic enzymes contribute to extracellular matrix degradation and amplification of the inflammatory response. *T. denticola* is an important component of the subgingival biofilm and plays a central role in oral dysbiosis and periodontal disease progression [92]. Among its principal virulence factors is dentilisin, a protease complex that facilitates nutrient acquisition, bacterial coaggregation, and immune evasion [93]. In addition, *T. denticola* promotes alveolar bone destruction by inhibiting osteogenic cell differentiation and stimulating the production of pro-inflammatory cytokines [94].

Together with *P. gingivalis*, *T. forsythia* and *T. denticola* constitute the classical red complex, which is strongly associated with severe chronic periodontitis [95].

*Streptococcus* is a genus of Gram-positive bacteria that plays an essential role in the early stages of oral biofilm formation. Species such as *Streptococcus mutans* are major contributors to dental caries through acid production and biofilm formation, leading to enamel demineralization [96]. Furthermore, early colonization by streptococci facilitates the subsequent attachment of periodontal pathogens within gingival pockets, thereby promoting the maturation of pathogenic biofilms and progression of periodontal disease [97].

Emerging evidence suggests that *T. forsythia* contributes to chronic periodontal inflammation that may extend beyond the oral cavity and promote systemic inflammation and neuroinflammatory processes [98]. Several studies have identified *T. forsythia* in patients with AD, supporting a potential association between periodontal infection and cognitive decline [99], 100]. In addition, the severity of periodontal disease has been correlated with the degree of cognitive impairment, suggesting that chronic oral inflammation may exacerbate neurodegenerative processes [101].

Similarly, *T. denticola* forms part of a consortium of periodontal pathogens that collectively contribute to systemic inflammation and neurodegeneration [102]. Current evidence indicates that persistent colonization by *T. denticola* may promote inflammatory pathways associated with both AD and PD, although the precise molecular mechanisms remain incompletely understood.

Interactions between *Streptococcus* species and neuronal cells have also attracted increasing attention. Experimental studies suggest that certain streptococcal species may induce neuronal injury and cell death through inflammatory and immune-mediated mechanisms, potentially contributing to long-term neurodegenerative changes [103]. Nevertheless, the relationship between *Streptococcus* species and neurodegenerative diseases remains incompletely understood and continues to be investigated, particularly with respect to neuroinflammatory and autoimmune mechanisms [103], 104].

Collectively, these findings suggest that *T. forsythia*, *T. denticola*, and selected *Streptococcus* species contribute to neurodegeneration primarily through chronic periodontal inflammation, systemic immune activation, and persistent neuroinflammatory signaling. Although the available evidence is less extensive than that for *P. gingivalis*, these microorganisms represent important components of the oral dysbiotic community associated with neurodegenerative disease.

#### *F. nucleatum* and *P. intermedia*

*Fusobacterium nucleatum* (*F. nucleatum*) is a Gram-negative, obligate anaerobic, spindle-shaped bacterium that is commonly found in the human oral cavity [105]. It is an opportunistic pathogen associated with a wide range of infectious diseases, and its role in gastrointestinal disorders has received increasing attention, particularly in the context of periodontal disease. *F. nucleatum* is widely recognized as a bridging organism because it coaggregates with both early and late bacterial colonizers, thereby facilitating the maturation of pathogenic dental biofilms and promoting the development of gingivitis and periodontitis [106].

*Prevotella intermedia* (*P. intermedia*) is also a Gram-negative, obligate anaerobic, short rod-shaped bacterium that colonizes the oral mucosa [107]. It belongs to the orange complex of periodontal microorganisms and is moderately associated with periodontal disease, functioning primarily as a late colonizer of the dental biofilm [107], 108]. *P. intermedia* is frequently detected in the subgingival plaque of patients with severe chronic periodontitis [108], 109].

An increasing body of evidence supports an association between chronic periodontitis and cognitive impairment, particularly AD [110], [111], [112], [113]. Elevated abundances of both *F. nucleatum* and *P. intermedia* have been reported in the oral microbiomes of patients with AD [98], [110], [111], [112]. Furthermore, two longitudinal retrospective cohort studies demonstrated that elevated serum antibody titers against periodontal pathogens preceded the clinical diagnosis of cognitive decline and AD, suggesting that chronic periodontal infection may occur early in disease development [114], 115].

Experimental evidence further supports a potential role for these microorganisms in neurodegeneration. Both *in vitro* and *in vivo* studies have investigated the effects of *F. nucleatum* [116] and *P. intermedia* [117] on AD progression. Notably, L’Heureux et al. identified an increased abundance of *P. intermedia* in individuals with mild cognitive impairment carrying the APOE4 genotype, suggesting that this organism may be associated with increased genetic susceptibility to dementia [117].

Nevertheless, whether a causal relationship exists between periodontal disease and neurodegenerative disorders remains uncertain. Several studies have proposed that blood–brain barrier dysfunction associated with AD may facilitate the entry of periodontal pathogens and their virulence factors into the central nervous system, thereby promoting neurodegeneration [102], 118]. Although the mechanisms by which pathogens such as *T. denticola* and *P. gingivalis* reach the brain are being actively investigated [119], 120], evidence supporting a similar mechanism for *F. nucleatum* remains limited and requires further investigation [105].

Neuroinflammation is widely recognized as a central mechanism underlying AD pathogenesis, and periodontal pathogens may contribute to this process by sustaining chronic systemic inflammation [113], 118], 121]. Wu et al. demonstrated that *F. nucleatum*-induced periodontitis exacerbated AD pathology in mice through activation of microglia and associated morphological changes [68]. In contrast, Bahar et al. used *F. nucleatum*-infected mice as a control group when investigating *P. gingivalis*-induced periodontitis and concluded that *F. nucleatum* played a comparatively limited role in AD pathology [122]. Nevertheless, the authors also observed increased activation of microglia and astrocytes in *F. nucleatum*-infected animals, although these changes were less pronounced than those induced by *P. gingivalis*.

Laugisch et al. [123] also investigated the relationship between *F. nucleatum*, *P. intermedia*, and PD. Their findings emphasized the importance of chronic inflammation in the progression of both periodontal disease and neurodegeneration, demonstrating that disease severity correlated with the abundance of periodontal pathogens. In addition, PD severity was inversely associated with glucose-6-phosphate dehydrogenase (G6PD) activity in both serum and saliva.

Collectively, these findings suggest that increased abundances of *F. nucleatum* and *P. intermedia* may contribute to chronic systemic inflammation and neuroinflammation, representing a promising area for future investigation. However, current evidence remains insufficient to establish a direct causal relationship between these microorganisms and neurodegenerative diseases, and further mechanistic and longitudinal studies are required.

Future integration of oral microbiome profiling, host genetic susceptibility (e.g., APOE genotype), and nutritional phenotyping may enable precision prevention strategies tailored to individual neurodegenerative risk.

Overall, the available evidence indicates that several major periodontal pathogens contribute to neurodegenerative processes through distinct virulence factors and overlapping inflammatory pathways. A comparative summary of these microorganisms, their proposed mechanisms of action, associated neurodegenerative diseases, and the current level of evidence is presented in Table 1.

1. Factors Influencing the Oral Microbiome

**Table 1:** Major oral pathogens and their proposed contributions to neurodegeneration.

Oral microorganism	Major virulence factors	Neurodegenerative disorder(s) associated	Proposed mechanisms	Representative evidence discussed in this review
*Porphyromonas gingivalis*	Gingipains, LPS, fimbriae, biofilm formation	AD, PD	Periodontal inflammation; systemic dissemination of LPS; cytokine release (IL-1α, IL-1β); BBB disruption; microglial activation; amyloid-β accumulation; tau pathology	LPS detected in brains of infected mice; experimental and clinical studies linking periodontitis with AD pathology
*Aggregatibacter actinomycetemcomitans*	Leukotoxin, LPS (serotypes A–C)	AD	Activation of TLR2/TLR4 signaling; increased cytokine production; enhanced Aβ1–42 production; neuroimmune activation	Experimental hippocampal culture studies and mouse models
*Tannerella forsythia*	S-layer glycoproteins, sialidases (NanH)	AD	Chronic periodontal inflammation; epithelial invasion; contribution to neuroinflammation through persistent immune activation	Clinical associations between periodontal disease and cognitive decline
*Treponema denticola*	Dentilisin, proteolytic enzymes	AD, PD	Tissue invasion; biofilm formation; induction of pro-inflammatory cytokines; inhibition of osteogenic differentiation; contribution to systemic inflammation	Experimental studies and association with periodontal dysbiosis in neurodegenerative diseases
*Streptococcus mutans*	Glucosyltransferases, extracellular polysaccharides, acid production	Indirect association with neurodegeneration	Biofilm formation; promotion of oral dysbiosis; facilitation of colonization by periodontal pathogens; neuroinflammatory and autoimmune mechanisms (proposed)	Experimental evidence; direct causal relationship remains under investigation
*Fusobacterium nucleatum*	Adhesins, LPS, co-aggregation (“bridging” organism)	AD, PD	Promotion of chronic systemic inflammation; activation of microglia and astrocytes; contribution to the oral–gut–brain axis; association with disease severity	Animal models, observational studies, and longitudinal cohort studies
*Prevotella intermedia*	LPS, proteolytic enzymes	AD; possible association with PD	Late colonization of periodontal biofilm; chronic inflammation; possible interaction with APOE4 genotype; contribution to neuroinflammatory processes	Clinical observational studies and genetic association studies

The composition of one’s oral microbiome is also modulated by numerous intrinsic and extrinsic factors (Table 2).

**Table 2:** Intrinsic and extrinsic factors modulating the oral microbiome and their biological consequences.

Factor	Effect on the oral microbiome	Physiological changes	Potential consequences
Diet (high sugar and fermentable carbohydrates)	Promotes dysbiosis	Enrichment of acidogenic bacteria (*Streptococcus mutans*), reduced plaque pH	Dental caries, enamel demineralization, disruption of oral microbial homeostasis
Diet rich in fiber	Supports microbial diversity	Increased diversity of the plaque microbiome	Maintenance of oral microbial homeostasis
Dietary nitrates	Favors beneficial microbiota	Enrichment of nitrate-reducing bacteria; increased nitric oxide (NO) production	Improved oral homeostasis and vascular health
Adequate intake of vitamins, unsaturated fatty acids, and antioxidants	Supports host defense	Improved antioxidant capacity and regulation of neuroinflammatory pathways	Reduced oxidative stress and maintenance of neuronal function
Polyphenols	Modulate oral biofilms	Inhibition of *Streptococcus mutans* growth, reduced bacterial adhesion and biofilm formation	Prevention of dental caries, maintenance of oral ecosystem balance
Probiotics/postbiotics	Enhance host resilience	Activation of Nrf2 signaling, improved antioxidant defense	Reduced periodontal inflammation and restoration of redox balance
Stress	Alters oral microbial homeostasis	Changes in host physiological balance and immune regulation	Increased susceptibility to dysbiosis
Alcohol consumption and xerogenic medications	Modify salivary environment	Reduced salivary flow and altered saliva composition	Changes in microbial composition and increased dysbiosis risk
Good oral hygiene	Maintains eubiosis	Reduction of dental plaque and pathogenic biofilm maturation	Stable oral microbiome and lower periodontal disease risk
Poor oral hygiene	Promotes dysbiosis	Biofilm accumulation and maturation into pathogenic communities	Increased risk of periodontitis
Antiseptic mouthwashes	Reduce microbial diversity	Depletion of beneficial bacteria, including nitrate-reducing species	Potential impairment of NO production and elevated blood pressure
Smoking	Promotes dysbiosis	Enrichment of *Fusobacteria*, *Prevotella*, and *Veillonella*; altered microbial functional pathways	Increased risk of periodontitis and chronic inflammation
Reduced salivary flow (xerostomia)	Disrupts microbial balance	Overgrowth of aciduric and pathogenic microorganisms	Increased susceptibility to caries and periodontal disease
Host genetics and immune response	Influences microbial selection	Individual variation in saliva composition and mucosal receptor profiles	Altered susceptibility to periodontal dysbiosis
Age and hormonal changes	Modify microbial composition	Hormone-associated increase in *Prevotella intermedia*; altered gingival environment	Increased susceptibility to gingivitis and periodontal disease
Diabetes mellitus	Creates a pro-inflammatory oral environment	Increased salivary glucose and enrichment of pathogenic communities	Greater risk of periodontitis
Broad-spectrum antibiotics	Disturb oral microbial ecology	Reduced microbial richness and overgrowth of opportunistic organisms (e.g., *Candida*)	Loss of colonization resistance and transient dysbiosis

### Factors influencing the oral microbiome

Diet is one of the principal factors shaping the oral microbiome. Frequent consumption of sugars and fermentable carbohydrates promotes the growth of acidogenic bacteria, particularly *S. mutans*, thereby lowering oral pH and increasing the risk of dental caries. In contrast, diets rich in dietary fiber support greater microbial diversity within dental plaque [124], 125]. Dietary nitrates, abundant in leafy green vegetables, enrich nitrate-reducing bacteria, thereby enhancing nitric oxide production and contributing to oral homeostasis [18].

Adequate intake of vitamins, unsaturated fatty acids, and antioxidants contributes to oral and systemic health and may reduce the risk of chronic inflammatory diseases [126].

B-group vitamins play essential roles in nervous system function. Thiamine (vitamin B1) participates in acetylcholine synthesis; riboflavin (vitamin B2) supports mitochondrial function and antioxidant defense; niacin (vitamin B3) serves as a precursor of nicotinamide adenine dinucleotide (NAD+), which is essential for neuronal metabolism; pyridoxine (vitamin B6) regulates homocysteine metabolism; biotin (vitamin B7) participates in fatty acid synthesis; folate (vitamin B9) contributes to methylation reactions and homocysteine regulation; and cobalamin (vitamin B12) is required for myelin synthesis and maintenance. Deficiency of these vitamins has been associated with peripheral neuropathy, muscle weakness, cognitive decline, and memory impairment [127].

Fat-soluble vitamins also play important roles in neurological health. Vitamin D regulates oxidative stress, neuroinflammation, and neuronal differentiation and maturation, and vitamin D deficiency has been associated with increased amyloid-β accumulation [128].

Omega-3 and omega-6 polyunsaturated fatty acids participate in the regulation of oxidative stress and serve as precursors for lipid mediators, including prostaglandins and leukotrienes [116]. Oxidative stress has been implicated in the pathogenesis of AD, where excessive reactive oxygen species promote lipid peroxidation, protein oxidation, DNA damage, and tau hyperphosphorylation [126].

Polyphenols are naturally occurring bioactive compounds characterized by one or more phenolic rings and are widely distributed in fruits, vegetables, tea, cocoa, berries, and numerous other plant-derived foods. They have attracted considerable interest because of their antioxidant, antibacterial, anti-inflammatory, and microbiome-modulating properties. Increasing evidence suggests that polyphenols may contribute to the prevention of chronic inflammatory diseases, including oral diseases, by maintaining microbial homeostasis and limiting pathogen colonization [129].

In dental caries, polyphenols have been extensively investigated for their antibacterial properties, particularly their ability to inhibit bacterial growth, adhesion, and biofilm formation. Dental caries results from disruption of the oral microbial ecosystem, with *S. mutans* serving as the principal cariogenic species. *S. mutans* produces extracellular polysaccharides that facilitate the formation of a highly structured three-dimensional biofilm [130]. Fermentation of dietary sugars generates organic acids that lower biofilm pH and promote enamel and dentin demineralization [131], 132].

Numerous studies have demonstrated that polyphenols inhibit the growth of *S. mutans*. Babaeekhou and Ghane reported that ginger extracts exhibit pronounced antibacterial activity against *S. mutans* and *S. sobrinus* [133]. Other polyphenol-rich preparations, including tart cherry, myrtle extract, and Chilean propolis, inhibit bacterial adhesion and interfere with biofilm formation [134], 135]. Clinical evidence also supports their preventive potential. In one *in vivo* study involving saliva and dental biofilm samples from 75 healthy individuals, a polyphenol-containing mouthwash significantly reduced the abundance of bacterial taxa associated with oral disease, regardless of dietary sugar intake [136].

Overall, current evidence suggests that polyphenols contribute to the maintenance of oral microbial homeostasis and represent promising adjunctive agents for preserving oral health. Nevertheless, additional mechanistic and clinical studies are required to clarify their interactions with oral microorganisms and host tissues.

Nuclear factor erythroid 2-related factor 2 (Nrf2) has emerged as a promising therapeutic target in periodontitis because of its central role in regulating oxidative stress and inflammation [137]. Nrf2 is a transcription factor that activates antioxidant response element (ARE)-dependent genes involved in cellular antioxidant defense and cytoprotection [138]. During periodontitis, excessive production of reactive oxygen species (ROS) contributes to tissue destruction, whereas impaired Nrf2 signaling is associated with reduced antioxidant capacity and enhanced inflammatory responses [139].

Recent studies suggest that probiotics and postbiotics activate Nrf2 signaling, thereby enhancing antioxidant defenses and reducing periodontal inflammation. These agents may therefore serve as adjuncts to conventional mechanical periodontal therapy by restoring redox balance and improving clinical outcomes [140]. However, further *in vivo* and clinical studies are required to clarify the molecular mechanisms of Nrf2 activation and to optimize their formulation, dosage, and long-term efficacy.

Psychological stress alters physiological homeostasis and has been associated with changes in oral microbial composition through neuroendocrine and immune-mediated mechanisms [141].

Alcohol consumption and xerogenic medications reduce salivary flow and alter salivary composition, thereby influencing the structure of the oral microbiome.

Oral hygiene practices strongly influence microbial composition. Regular tooth brushing and interdental cleaning disrupt dental biofilms and reduce microbial burden, whereas inadequate oral hygiene promotes biofilm maturation and dysbiosis. Antiseptic mouthwashes may transiently reduce microbial diversity and, when used excessively, may suppress beneficial nitrate-reducing bacteria, potentially contributing to elevated blood pressure [18].

Smoking exerts profound effects on the oral microbiome. Smokers generally exhibit increased microbial diversity together with enrichment of pathogenic taxa, including *Fusobacterium*, *Prevotella*, and *Veillonella*. Smoking-associated dysbiosis contributes to chronic inflammation and substantially increases the risk of periodontitis [142].

Salivary factors, including flow rate, pH, and immunoglobulin content, are major determinants of microbial composition. Reduced salivary flow (xerostomia) promotes microbial imbalance and overgrowth of acid-tolerant and pathogenic microorganisms.

Host genetics and immune function also influence oral microbial composition. Genetic variation in immune-response genes affects susceptibility to periodontal dysbiosis despite similar microbial exposures [143].

Age and hormonal changes further modulate the oral microbiome. Hormonal fluctuations during puberty and pregnancy alter gingival physiology and microbial composition, including increased abundance of *P. intermedia* during pregnancy-associated gingivitis [124]. Systemic diseases such as diabetes mellitus also promote a pro-inflammatory oral environment characterized by elevated salivary glucose concentrations, favoring the growth of pathogenic microorganisms.

Antibiotics and other medications transiently disrupt the oral microbiota. Broad-spectrum antibiotics reduce microbial diversity and may permit opportunistic organisms, particularly *Candida* species, to overgrow.

Overall, the oral microbiome is highly dynamic and responds continuously to diet, oral hygiene, lifestyle factors, smoking, medications, host genetics, aging, and systemic health. Maintenance of good oral hygiene, a balanced diet, and avoidance of tobacco are essential for preserving a stable, health-associated oral microbiome and preventing dysbiosis [18], 124], 125].

### Phenolic acids, microglial polarization, and autophagy in cognitive impairment

Recent clinical trials and high-quality *in vivo* studies have provided increasingly robust evidence supporting the efficacy of specific classes of polyphenols in neuronutrition and oral health. A randomized controlled trial published in 2022 demonstrated that daily supplementation with 500 mg of resveratrol, a stilbene-class polyphenol, for 26 weeks significantly reduced plasma concentrations of IL-6 and TNF-α in patients with mild-to-moderate AD compared with placebo. This effect was accompanied by a reduction in cerebrospinal fluid biomarkers of neuroinflammation [144]. Similarly, epigallocatechin gallate (EGCG), the principal catechin found in green tea, reduced Aβ1–42 oligomerization and improved composite cognitive scores in phase II clinical trials over 36 weeks, supporting its potential as a disease-modifying neuronutritional agent [145]. In addition, a 2023 clinical study demonstrated that nano-emulsified curcumin significantly reduced *P. gingivalis*-associated gingival inflammation while overcoming the poor bioavailability traditionally associated with curcumin formulations [146].

The pharmacological properties of individual polyphenol classes differ considerably with respect to their antimicrobial specificity and systemic anti-inflammatory effects. Flavonoids, including quercetin and kaempferol, primarily inhibit *P. gingivalis* adhesion and biofilm formation by interfering with interactions between FimA fimbriae and host receptors. Phenolic acids, including rosmarinic acid and chlorogenic acid, exhibit broader antibacterial activity against *T. denticola* and *F. nucleatum* through disruption of bacterial membrane potential and inhibition of quorum-sensing pathways. In contrast, stilbenes such as resveratrol preferentially modulate systemic NF-κB and SIRT1 signaling pathways, thereby reducing downstream cytokine production without exerting pronounced direct antibacterial effects. Tannins, including ellagic acid and punicalagin, demonstrate potent anti-biofilm activity against multiple members of the periodontal red complex [147].

Despite these promising biological activities, the clinical translation of polyphenol-based neuronutritional strategies remains limited by poor bioavailability and restricted penetration into brain tissue. Curcumin and quercetin undergo extensive hepatic conjugation and intestinal glucuronidation, resulting in only nanomolar concentrations within the central nervous system. Advanced drug-delivery systems – including lipid nanoparticles, phospholipid complexes, and cyclodextrin encapsulation – have therefore been developed to improve intestinal absorption and blood–brain barrier penetration. Resveratrol and EGCG exhibit comparatively greater central nervous system bioavailability because of their lower molecular weight and reduced dependence on P-glycoprotein transport mechanisms [148]. These pharmacokinetic limitations highlight the importance of formulation science for translating promising experimental findings into clinically effective neuronutritional interventions.

Among dietary bioactive compounds, phenolic acids – including rosmarinic acid, chlorogenic acid, ferulic acid, and caffeic acid have emerged as particularly attractive multi-target agents for both vascular and neurodegenerative cognitive impairment. Vascular cognitive impairment is characterized by endothelial dysfunction, oxidative stress, and cerebrovascular inflammation, which reduce cerebral blood flow and promote white matter injury. Chlorogenic acid activates endothelial nitric oxide synthase (eNOS) through AMPK phosphorylation, thereby improving endothelial function. Ferulic acid scavenges peroxynitrite and superoxide radicals, protecting the vascular endothelium, whereas rosmarinic acid inhibits matrix metalloproteinases (MMP-2 and MMP-9), thereby reducing blood–brain barrier degradation associated with chronic neuroinflammation [149], 150].

Regulation of microglial polarization represents another important mechanism through which phenolic acids may influence neurodegeneration. Caffeic acid and caffeic acid phenethyl ester (CAPE) suppress pro-inflammatory M1 microglial polarization by inhibiting TLR4-mediated NF-κB nuclear translocation and reducing the expression of inducible nitric oxide synthase (iNOS), cyclooxygenase-2 (COX-2), and the pro-inflammatory cytokines IL-1β, IL-6, and TNF-α [151]. Conversely, ferulic acid and rosmarinic acid promote the anti-inflammatory M2 phenotype through activation of peroxisome proliferator-activated receptor-γ (PPAR-γ) and increased secretion of IL-10 and transforming growth factor-β (TGF-β) [152]. This coordinated suppression of M1 activation together with promotion of M2 polarization is particularly relevant within the oral–brain axis, where chronic peripheral cytokine signaling arising from periodontal disease persistently shifts the central nervous system immune environment toward a pro-inflammatory phenotype.

Phenolic acids also exert neuroprotective effects through modulation of autophagy, a cellular quality-control pathway essential for the removal of misfolded proteins and dysfunctional organelles. Impaired mitophagy and defective autophagic clearance of amyloid-β and α-synuclein aggregates are recognized as central pathological mechanisms in both AD and PD. Resveratrol and EGCG stimulate autophagy through activation of the AMPK–mTOR and SIRT1–FOXO3 signaling pathways, thereby restoring autophagic flux and enhancing lysosomal clearance of neurotoxic protein aggregates [153]. Chlorogenic acid increases beclin-1 expression and promotes LC3-II conversion in neuronal cells exposed to lipopolysaccharide, directly linking endotoxin-induced stress associated with periodontal pathogens to an autophagic rescue response mediated by dietary phenolic compounds [154].

**Figure 2:**
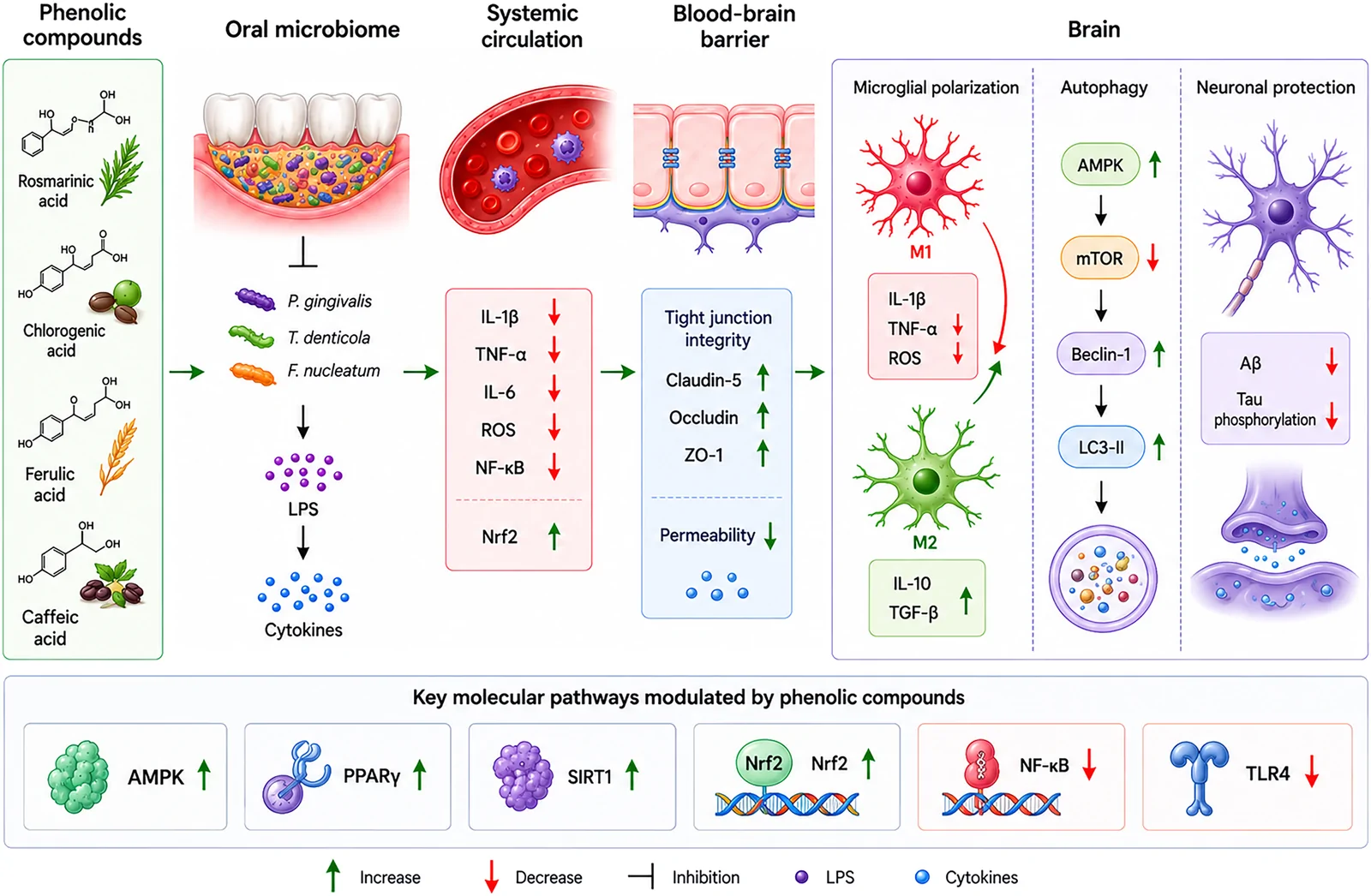
Multi-target mechanisms of phenolic compounds along the oral–brain axis.

The multiple mechanisms through which phenolic compounds modulate oral dysbiosis, neuroinflammation, vascular dysfunction, and neuronal resilience are summarized in Figure 2.

Phenolic compounds, including **rosmarinic acid, chlorogenic acid, ferulic acid, and caffeic acid**, modulate multiple biological pathways linking oral health to brain function. By suppressing key periodontal pathogens (*P. gingivalis, T. denticola,* and *F. nucleatum*), they reduce LPS release and systemic production of pro-inflammatory cytokines (IL-1β, TNF-α, and IL-6). These effects are associated with activation of Nrf2, inhibition of NF-κB signaling, preservation of blood–brain barrier integrity through increased expression of claudin-5, occludin, and ZO-1, and reduced permeability. Within the central nervous system, phenolic compounds promote microglial polarization toward the anti-inflammatory M2 phenotype and stimulate AMPK-dependent autophagy via inhibition of mTOR and upregulation of Beclin-1 and LC3-II. Collectively, these mechanisms attenuate neuroinflammation, reduce amyloid-β accumulation and tau hyperphosphorylation, and support neuronal protection through coordinated modulation of the oral–brain axis.

Collectively, these findings suggest that phenolic acids function as a mechanistic bridge between oral microbiome homeostasis and neuronal resilience. Their pleiotropic actions integrate vascular protection, modulation of neuroimmune signaling, regulation of microglial polarization, and maintenance of autophagic proteostasis, highlighting their considerable therapeutic potential within precision neuronutrition strategies.

## Conclusions

The convergence of neurology, oral medicine, microbiology, and neuronutrition represents an emerging interdisciplinary framework for understanding the complex interactions between oral health and neurodegenerative diseases. Current evidence indicates that chronic oral dysbiosis and periodontitis contribute to persistent systemic inflammation, immune dysregulation, blood–brain barrier dysfunction, and alterations in the oral–gut–brain axis, thereby creating biological conditions that may promote neuroinflammation and accelerate the progression of neurodegenerative disorders. Although definitive causal relationships remain to be established, accumulating epidemiological, experimental, and mechanistic evidence supports the oral microbiome as a potentially modifiable contributor to brain health.

This review highlights that oral microorganisms and their virulence factors influence multiple interconnected pathways, including activation of innate immune signaling, cytokine production, oxidative stress, microglial polarization, disruption of blood–brain barrier integrity, and impaired proteostatic mechanisms. Together, these processes provide a biologically plausible framework linking chronic periodontal inflammation with the pathogenesis of disorders such as AD and PD. At the same time, the available evidence suggests that neuronutritional interventions including polyphenols, dietary nitrates, omega-3 fatty acids, vitamins, probiotics, and postbiotics may complement conventional oral and neurological care by promoting oral microbial homeostasis, attenuating systemic inflammation, preserving BBB integrity, and enhancing neuronal resilience.

From a clinical perspective, closer collaboration among neurologists, dentists, periodontists, and neuronutrition specialists may facilitate earlier identification of individuals at increased risk of neurodegenerative disease and support the development of integrated preventive strategies. Routine assessment of oral health may represent an important component of comprehensive brain health evaluation, particularly in aging populations and individuals at increased risk of neurodegenerative diseases. Despite the growing body of evidence, several important limitations should be acknowledged. Most available studies are observational, cross-sectional, or based on experimental animal models, limiting the ability to establish direct causal relationships between oral dysbiosis and neurodegenerative diseases. Considerable heterogeneity in microbiome profiling methodologies, periodontal disease definitions, study populations, and cognitive outcome measures further complicates comparisons across studies. In addition, although neuronutritional interventions show considerable promise, clinical evidence remains limited, and relatively few large randomized controlled trials have evaluated their long-term effects on cognitive outcomes.

Future research should prioritize well-designed longitudinal cohort studies, mechanistic investigations, and randomized clinical trials to clarify causal relationships, identify reliable microbiome-derived biomarkers, and determine whether targeted modulation of the oral microbiome can alter the onset or progression of neurodegenerative diseases. Integrating advances in microbiome science, neuroimmunology, precision nutrition, and systems medicine will be essential for developing evidence-based preventive and therapeutic strategies. Collectively, the findings summarized in this review support the concept that maintaining oral microbial homeostasis may represent a promising and potentially modifiable target within multidisciplinary approaches aimed at preserving cognitive function and promoting healthy brain aging.
